# Palliative and end-of-life care in care homes: protocol for codesigning and implementing an appropriate scalable model of Needs Rounds in the UK

**DOI:** 10.1136/bmjopen-2021-049486

**Published:** 2021-02-22

**Authors:** Aisha Macgregor, Alasdair Rutherford, Brendan McCormack, Jo Hockley, Margaret Ogden, Irene Soulsby, Maisie McKenzie, Karen Spilsbury, Barbara Hanratty, Liz Forbat

**Affiliations:** 1 Faculty of Social Sciences, University of Stirling, Stirling, UK; 2 Divisions of Nursing, Occupational Therapy & Arts Therapies, Queen Margaret University, Edinburgh, UK; 3 Usher Institute, College of Medicine and Veterinary Medicine, The University of Edinburgh, Edinburgh, UK; 4 School of Healthcare, University of Leeds, Leeds, UK; 5 Population Health Sciences Institute, Newcastle University, Newcastle upon Tyne, UK

**Keywords:** adult palliative care, protocols & guidelines, geriatric medicine, palliative care

## Abstract

**Introduction:**

Palliative and end-of-life care in care homes is often inadequate, despite high morbidity and mortality. Residents can experience uncontrolled symptoms, poor quality deaths and avoidable hospitalisations. Care home staff can feel unsupported to look after residents at the end of life. Approaches for improving end-of-life care are often education-focused, do not triage residents and rarely integrate clinical care. This study will adapt an evidence-based approach from Australia for the UK context called ‘Palliative Care Needs Rounds’ (Needs Rounds). Needs Rounds combine triaging, anticipatory person-centred planning, case-based education and case-conferencing; the Australian studies found that Needs Rounds reduce length of stay in hospital, and improve dying in preferred place of care, and symptoms at the end of life.

**Methods and analysis:**

This implementation science study will codesign and implement a scalable UK model of Needs Rounds. The Integrated Promoting Action on Research Implementation in Health Services (i-PARIHS) framework will be used to identify contextual barriers and use facilitation to enable successful implementation. Six palliative care teams, working with 4–6 care homes each, will engage in two phases. In phase 1 (February 2021), stakeholder interviews (n=40) will be used to develop a programme theory to meet the primary outcome of identifying what works, for whom in what circumstances for UK Needs Rounds. Subsequently a workshop to codesign UK Needs Rounds will be run. Phase 2 (July 2021) will implement the UK model for a year. Prospective data collection will focus on secondary outcomes regarding hospitalisations, residents’ quality of death and care home staff capability of adopting a palliative approach.

**Ethics and dissemination:**

Frenchay Research Ethics Committee (287447) approved the study. Findings will be disseminated to policy-makers, care home/palliative care practitioners, residents/relatives and academic audiences. An implementation package will be developed for practitioners to provide the tools and resources required to adopt UK Needs Rounds.

**Registration details:**

Registration details: ISRCTN15863801.

**Strengths and limitations of this study**

- Using implementation science will improve the chances of Needs Rounds being successfully implemented across the sites.
- Data collection techniques will flexibly use video interviews and virtual workshops to accommodate lockdown and social distancing measures.
- Care home staff collection of resident data will prevent the need for research staff to access personal data or enter care homes when there are concerns about COVID-19 transmission.

## Introduction

Care home residents (hereafter referred to as residents) are an ageing population and are often frail with multiple complex health needs.^1 2^ Older people’s care homes in the UK experience high mortality rates, with between 26%^3^ and 56%^1^ of care home residents dying in the first year of admission. Care homes, including those with and without registered nurses (RNs), increasingly look after older people with complex multiple morbidities.^4^ Projections show that if current trends continue, care homes will be the most common place of death by 2040.^5^

Residents often experience inadequate end-of-life care as a result of avoidable hospitalisations,^6^ unmanaged symptoms, poor anticipatory/advance care planning^7^ and communication barriers.^8 9^ Staff report difficulties with interdisciplinary working,^10^ and low confidence and capability^7^ in managing symptoms and identifying when people are dying, deficiencies exacerbated by workforce issues, including staff shortages, high turnover^11 12^ and time restrictions.^13^

Residents often have multiple admissions to hospital prior to their death,^6^ with some admissions potentially preventable.^14^ Hospitalisations are costly and may prompt futile interventions that can cause distress to residents and family members.^15^ Developing evidence-based approaches to support older people at the end of life, and reducing avoidable and often detrimental admissions to acute care, must therefore be a priority.

A systematic review of end-of-life care in UK care homes calls for the extension of specialist palliative care in care homes.^10^ Key issues reported include staff struggling to identify when residents are approaching end of life and unanticipated deterioration impacting decision-making about hospitalisations. Anticipatory/advance care planning training needs were also highlighted, with staff frequently avoiding these conversations due to lacking confidence and knowledge. The need to strengthen interdisciplinary working to address reactive decision-making and improve understanding about who is responsible for discussions about end-of-life care was also described.

Providing end of life support to care homes is a growing area of service development. Current approaches such as ECHO (Extension of Community Healthcare Outcomes),^16^ Gold Standard Framework (GSF),^17^ Macmillan’s education for carers ‘Foundations in Palliative Care’, Six Steps to Success,^18^ PACE (Palliative Care Across Europe)^19^ and the Namaste programme^20^ offer staff training, yet rarely facilitate evidence-based clinical input for people diagnosed as dying. Both the GSF^21^ and PACE^22^ recommend monthly interdisciplinary meetings, although this is often variable in practice. Furthermore, such interventions often fail to change behaviour.^23 24^

This study aims to address recognised deficits in the provision of palliative and end-of-life care in care homes, by using an implementation science approach to alter practice and embed a promising approach developed in Australia called ‘Palliative Care Needs Rounds’ (Needs Rounds). Needs Rounds improve palliative and end-of-life care by reducing unnecessary hospitalisations, increasing staff knowledge and capability, improving anticipatory/advance care planning and strengthening interagency working.

### Intervention description

Needs Rounds are monthly hour-long triage meetings focused on 8–10 residents in each care home who are at risk of dying without a plan in place. A checklist^25^ is used to identify the most appropriate residents to discuss. Needs Rounds are chaired by a palliative care specialist and attended by care home staff who discuss each resident’s physiological, psychosocial and spiritual needs. This instigates a number of tailored actions, which are always personalised for the individual, including medication reviews, case conferences, anticipatory/advance care planning and referrals to relevant specialists.

Needs Rounds have a strong evidence base to support their uptake. Research in Australia demonstrated a number of positive outcomes. Needs Rounds reduced the length and number of hospitalisations,^26 27^ increased resident dying in their preferred place,^26^ with dignity, compassion and comfort,^28^ enabled staff to normalise death and dying,^29^ and improved workforce confidence.^28^

### Aims and objectives

This study aims to codesign and implement an appropriate scalable UK model of Needs Rounds, which offers specialist palliative care outreach to care homes, in order to improve the lives and deaths of residents. The objectives are:

### Implementation

1. Codesign a UK version of Needs Rounds, which is responsive to the different (macro, meso and micro) contextual characteristics of the UK care home sector.
2. Implement the adapted model of care, assess feasibility, acceptability and effectiveness, and ultimately propose how the model of care can be further refined and adopted in the UK context, to reap the benefits demonstrated in the Australian work.

### Intervention

1. Determine the transferability of the core elements of the Needs Rounds intervention in a UK context.
2. Delineate the mechanisms of action that enable more effective palliative and end-of-life care practices to be realised in UK care homes.
3. Identify the relationships between (1) the mechanisms of action embedded in Needs Rounds, (2) how these mechanisms function in different care home contexts and (3) the outcomes arising for different stakeholders and parts of the care system.

### Process evaluation

1. Document the outcomes of UK Needs Rounds on hospitalisations (including costs), quality of death/dying and staff capability.
2. Assess and report the perspectives of care home residents/relatives/staff and palliative care staff on using UK Needs Rounds.
3. Map the UK Needs Rounds fit for services not engaged in the implementation study.
4. Identify the outcomes, impact and experience of patient/public involvement (PPI) in the study.

## Methods and analysis

This pragmatic critical-realist implementation study^30^ will use the Integrated Promoting Action on Research Implementation in Health Services (i-PARIHS) framework^31 32^ in six case studies. Each case comprises a specialist palliative care team working with 4–6 care homes each.

i-PARIHS represents an integrated approach, recognising that most implementation is complex, requiring attention to multiple factors simultaneously for an innovation to be successful. The development of theory is central to i-PAHRIS and enables effective implementation of research evidence into everyday practice.^33^ Programme theories explaining micro changes and transactions, such as working hypotheses or local theories of change, are explored to elucidate core concepts. From these, mid-range theories are developed that have greater explanatory potential to predict and plan for change across different settings.^33^ For this study, theories will be generated regarding (1) influential components of the UK context and (2) the mechanisms of how to implement Needs Rounds in care homes in order to deliver desired outcomes.

Facilitation is also critical for successful implementation.^31^ Facilitators are agents of change who lead or champion an innovation. Facilitators for each case will be identified during phase 1 data collection.^34^ Recognising the likelihood of high turn-over of staff in care homes, at least two key staff members (facilitators) will be identified for each care home.

### Target sites and population

Four case study sites will be in England and two in Scotland. Each site consists of a palliative care specialist service which will work with 4–6 care homes in their local area. Recruitment will be undertaken by the hospices with support from the research team and ENRICH (a national network for care home research) where necessary.

Purposive maximum variability sampling of specialist palliative care services will focus on recruiting a heterogeneous and information-rich sample to reflect, for example: urban/rural, service size, deprivation, cultural demographics, use of ECHO^35^ or other specialist palliative care input models, hospital at home services and care home support teams, national charity/independent management, funding models, and hospital transfer policies. These variables reflect the dominant contextual influences which are likely to impact how Needs Rounds are used in the UK.

Table 1 provides a breakdown of the inclusion criteria for each participant group. The qualitative sample (n=40 interviewees) seeks adequate depth and breadth to determine the implementation objective: ‘what works for whom under what circumstances’. Interview sample sizes are based on the Australian studies, and are congruent with accepted standards for qualitative data interpretation.^36^

**Table 1:** Sample and inclusion criteria

Participant	Inclusion criteria
Stakeholders (phases 1 and 2 interviews). n=40	Work for the specialist palliative care service or a care home (with or without registered nursing) in one of the six cases; or are a resident in one of the care homes; or are a relative of a care home resident in one of the six cases; or work in acute care impacted by hospitalised care home residents Willing to provide informed consent Have capacity to provide their own consent to participate Not engaged in any current safeguarding investigations.
Care homes (adopting Needs Rounds as their new standard of care). n=28–36	Located near to the specialist palliative care team Provide care to residents who have high clinical nursing/medical needs Willing to sign a memorandum of understanding with the research team, outlining resident demographics and health service use data, facilitate access to staff for interviews, and engagement in Needs Rounds A range of sizes (focusing primarily on larger care homes, following CQC data indicating lower quality in larger facilities),^46^ sole traders and large corporate provider, and with a range of funding models (NHS/social care and self-funded residents).
Residents (discussed at Needs Rounds in phase 2) Estimate of n=1500	Resident in a collaborating care home in one of the six case study locations An anticipated life-expectancy of less than 6 months At risk of dying without appropriate planning in place Experiencing inadequately managed bio-psychosocial symptoms Not engaged in any current safeguarding investigations Able to provide their own informed consent
Relatives (completing outcome measures in phase 2) n=300	The relative of a resident who was discussed in Needs Rounds Able to provide their own informed consent.
PPI evaluation n=15	Coinvestigator or a member of one of the case study sites Able to provide their own informed consent

CQC: Care Quality Commission; NHS: National Health Service

For the quantitative sample, the average size of the care homes is anticipated to be 52 beds, and the rate of emergency admission to hospital to be 0.173 per bed within the 4-month period,^37^ with an average of 9 admissions per care home. A sample of 30 care homes would allow change to be identified in the hospitalisation rate of 0.02 per bed (α=0.05, β=0.2, ΔSD=0.040). This is sufficient to detect a clinically meaningful change in the primary outcome (reduction of one hospitalisation per 4 month period in a typical care home) for the quantifiable data in phase 2.

### Project phases

#### Phase 1: codesigning Needs Rounds for the UK context

The purpose of phase 1 is to conduct theoretical modelling to generate programme theories about how Needs Rounds could be used. Programme theories will be developed by examining what elements of Needs Rounds would work, for whom, in what circumstances and why, in the UK context. Data will be collected through 40 semistructured individual or small-group interviews with key stakeholders from the six case study sites. Interviewees will be asked to describe their local context, such as services’ geography, policy, structure, funding and practice elements.

Contextual factors can significantly impact implementation; therefore, understanding and being responsive to these components are essential.^34^ Key contextual factors will be mapped out in the phase 1 interviews, drawing on the i-PAHRIS inner (micro and meso) and outer level (macro) contextual factors.^38^ The former includes individual and organisational components such as culture, leadership, experience of innovation, and absorptive capacity, while the latter examines the wider health system, including policy drivers, regulatory frameworks, and interorganisational networks and relationships. Services’ geography, policy, structure, funding and practice elements will then be refined in workshops to codesign Needs Rounds. Differences between the UK and Australia will be described.

**Figure 1:**
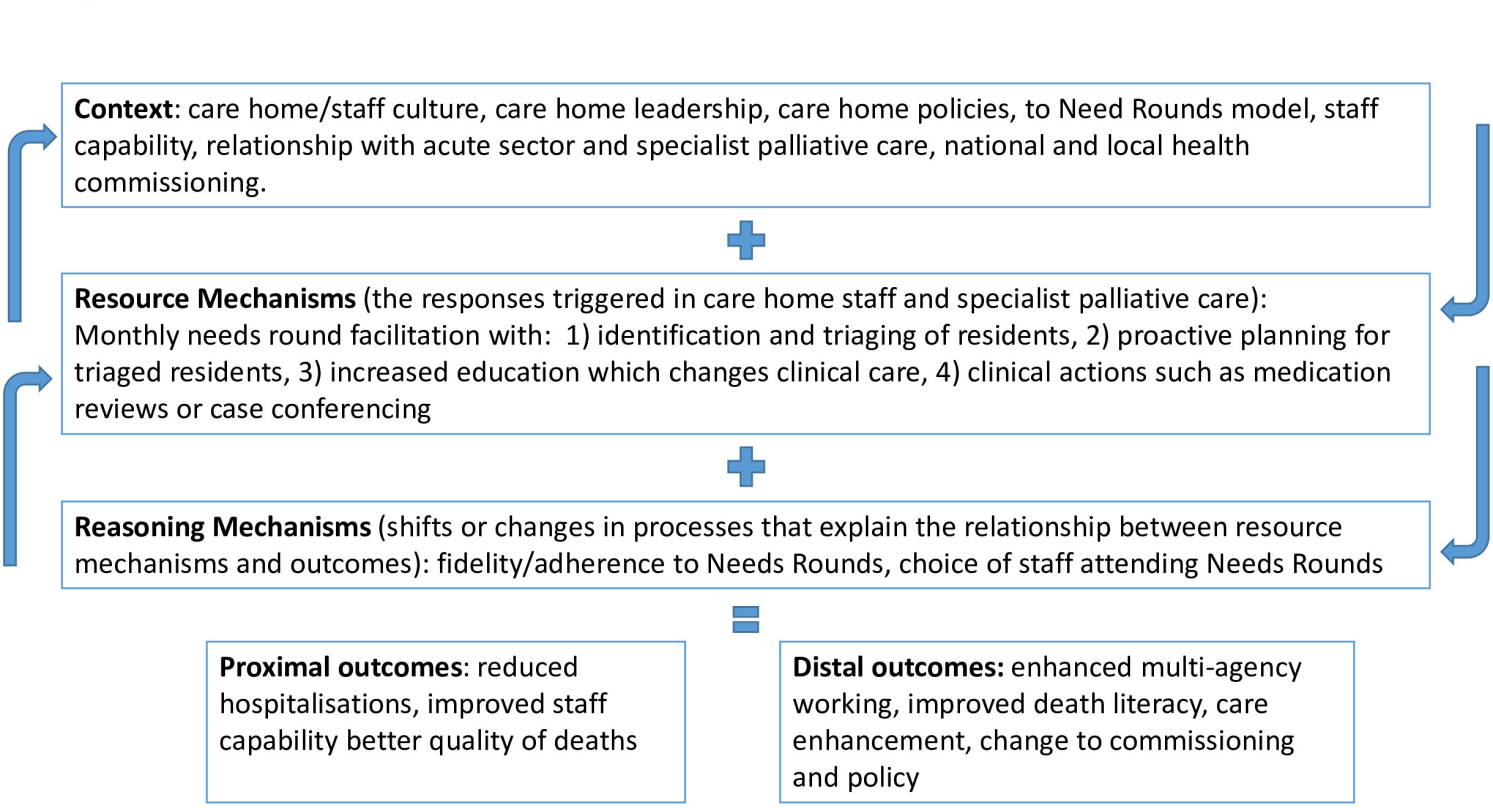
Contexts, mechanisms and outcomes derived from the Australian trial of Needs Rounds.

Relevant documentation (eg, service policies) will be collated to help develop realist theories regarding how implementation would work in practice and what might influence implementation in each case study site, to identify contextual factors, mechanisms and outcomes (CMO) (figure 1).

#### Phase 2: implementing and evaluating Needs Rounds

Phase 2 will test and refine the programme theories generated in phase 1, by implementing, adapting and evaluating UK Needs Rounds in the six case study sites. Interviews will be conducted after 4 months (capturing early adoption), 8 months (mid-range) and 12 months (longer term) of implementation. Interviews will collect prospective data on process and mechanisms of change and examine the CMOs/theories, acceptability, appropriateness, feasibility, implementation cost, coverage, sustainability and adherence.

Phase 2 will also incorporate a qualitative evaluation of the impact, outcomes and process of PPI throughout the study. A survey will be conducted with care homes not engaged in the implementation after the phase 2 workshop to garner feedback on the fit of the UK Needs Rounds approach for their service.

**Figure 2:**
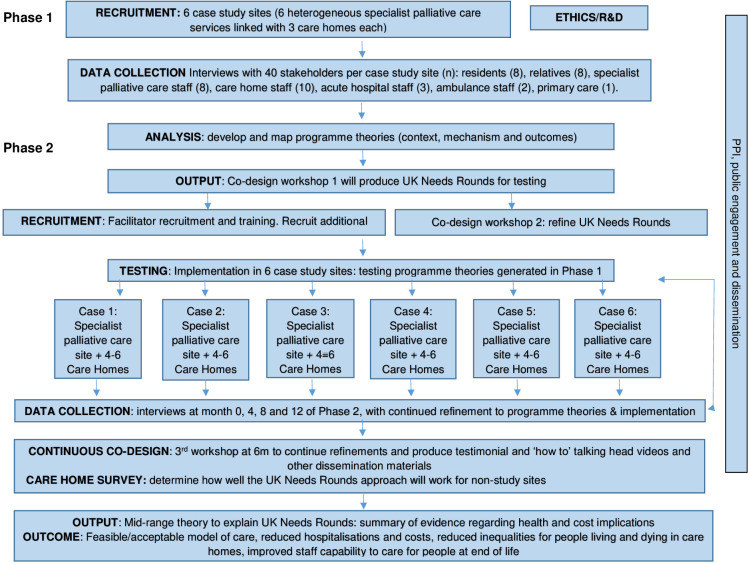
Study phases.

Key study phases are depicted in figure 2.

### Outcomes

The primary outcome is to determine *what works, for whom, in what circumstances,* for UK Needs Rounds. Secondary outcomes focus on health service use, alongside quality of death and dying, staff capability to adopt a palliative approach, and family perceptions of care. The core economic outcome is the total change in health and social care service costs that result from the intervention.

### Data collection

Data will be collected for each case from interviews and site documentation. Activity logs will also be generated, to capture time spent by all parties, and additional work generated beyond the Needs Rounds meeting. Environmental/contextual data will draw from conceptual work by Estabrook *et al*^39^ and i-PARIHS^34 38^ and be qualitative in nature to dynamically explore each care home’s culture. Interview topics will cover, for example, provider and funding types, leadership, culture, including attitudes towards residents and whether staff feel supported and valued, time/space, staff/resident turnover, introduction of new policies/procedures, and prioritisation of the intervention in workload.

Needs Rounds discussions will be audio-recorded. This will allow analysis of adaptations made by clinical teams for their local areas, alongside the breadth/depth/content of case-based education provided.

Staff capability of adopting a palliative approach (CAPA) will be assessed on a nine-item validated self-report questionnaire.^40^ CAPA has a unidimensional scale; higher scores indicate greater capacity. Internal consistency reliability is very high with a Cronbach’s alpha of 0.95, and split-half-reliability coefficient of 0.93.^40^ Self-complete measures will be taken at baseline from all nursing assistants/care assistants and RNs, and prospectively each month with staff attending Needs Rounds. Final assessment will be taken from all NAs and RNs following the 12-month trial period.

The Quality of Death and Dying Index^41^ (QODDI) will be completed by care home staff for each decedent resident prospectively during implementation. This 17-item questionnaire examines four domains: symptom control, preparation, connectedness and transcendence, on a 0–10 scale, where higher scores indicate a better experience. The Cronbach’s alpha for the QODDI total score is 0.89. Following correspondence with the scale’s originator confirming psychometric robustness of excluding items, one item on access to euthanasia will be removed, as this is not legal in the UK. The QODDI was designed for completion by relatives; however, staff are more likely to have seen the resident in the weeks prior to death so staff completion will result in more reliable and valid data.

Family perceptions of care will be captured from relatives of residents who are discussed at Needs Rounds, using the CANHELP lite.^42^ Twenty-two items from the second half of the questionniare will be used to collect family self-reported data of satisfaction with care. The Cronbach’s alpha for the total score is 0.88–0.94. A family will only be asked once to complete this measure, even if the resident is discussed at Needs Rounds more than once.

Resident data collection will focus on demographic information and health service use (table 2). Data will be collected from the care homes by care home staff. Training will be provided to ensure robust data collection and reporting. This will be summary level data to ensure anonymity.

**Table 2:** Resident data collection

Type	Variables
Demographic information	Age Ethnicity First language Charlson Comorbidity Index
Deaths	Number of deaths Preferred place of death Actual place of death
Health service use	Clinical role seen in primary care Hospital admissions: Name of hospital Duration of admission Specialty/ward Mode of transport to hospital
Assessments and interventions triggered by Needs Rounds	Physical assessments Clinical investigations, for example, blood/urine tests Referrals to other NHS services Changes in medicines Syringe drivers

Economic evaluation will be based on the intervention and health service cost elements identified in tables 3 and 4. The costs of hospitalisation will be constructed from resident-level data on length of stay collected by the care homes, and costed using the National Tariffs and hospital specific Patient Level Information and Costing System data for both England and Scotland. Total costs for the each of the pre and post periods will be calculated by summing these costs across all residents admitted to hospital from a given site; the benefit is the difference between prehealth and posthealth service-use costs.

**Table 3:** Intervention cost elements

Cost type	Cost detail	Measurement of costs
Direct costs	Intervention costs on-site: Staff time Travel Consumables and equipment Workshop costs	Included within the project budget and therefore directly recorded. Where appropriate additional detail will be collected directly from the care homes.
	Additional NHS staff time attending care home Additional prescriptions	Estimated in the SoECAT, with additional costs recorded by intervention staff as required.
	Costs of taxis to transfer residents to hospital	Collected from care home sites in a proforma through interviews.
Indirect costs	Wider additional costs incurred by the care home, including: Changes in their staffing Changes to facilities (ie, use of rooms), or overheads as a result of hosting the intervention.	These changes, and their associated costs, will be collected from care homes in a proforma through the interviews
Intangible costs	Inconvenience to staff, residents, family and carers as a result of the intervention.	These will not be measured directly, but will be explored in the qualitative interviews.

**Table 4:** Health service cost elements

Cost type	Cost detail	Measurement of costs
Direct costs	Costs of ambulance journeys	Estimated from the 2019/20 National Tariff Payment System.^47^
	Hospital stay cost	Hospital-specific Patient Level Information and Costing System data for England^48^ and Scotland^49^ on stay costs by age and gender to estimate a day rate to use in the hospital costing.
	Primary care usage	Collected from care home sites in a proforma through interviews
Indirect costs	Wider additional costs incurred by the care home, in connection with resident hospital admissions, including staffing, travel, equipment or facilities.	These costs will be collected from care homes in a proforma through the interviews
Intangible costs	Inconvenience to residents and their family/carers arising from hospitalisation	These will not be measured directly, but will be explored in the qualitative interviews in the main study.

### Analysis

Fidelity will be assessed through analysis of a random sample of 20% of all audio-recorded Needs Rounds to assess adherence to the agreed approach developed in the workshops. A three-tier scoring system will be adopted, of 1 (high adherence), 2 (moderate), 3 (low), with operational definitions for these scores developed prospectively as UK Needs Rounds are developed.

Transcripts of audio data and documentary evidence will be stored and organised using NVivo. Within and between case analysis will be conducted inductively, drawing on process tracing and constant comparative methods, respectively. Differences between the Australian context and the UK will be surfaced to facilitate detailed reporting on the specificity of the UK model to the local context. i-PARIHS will also be used to structure the analysis, and deductive analysis employed to refine the CMO theories. Thematic analysis will underpin the analytic approach, and follow the five-step process outlined by Braun and Clarke.^43^

CAPA, QODDI and CANHELP will be analysed with t-tests and presented using descriptive statistics, with statistical significance level set at p 0.05.

### Economic analysis: estimating the treatment effect of the intervention on health service outcomes

Data on the number and duration of hospitalisations for all care home residents will be captured for the 4 months prior to implementation, and in months 9–12 of delivery. This allows time for the intervention to be established and ensures that equivalent 4 month periods are compared with control for seasonality.

The treatment effect will be estimated as paired t-tests of the rate of hospitalisation, and number of hospital days, respectively. Multilevel regression modelling of the two outcome measures will be conducted, controlling for local area deprivation, sector of the care home, and other characteristics to describe the wider factors associated with the changes in the outcomes observed. A weighted least squares model of the outcomes will be estimated, with cases weighted by the number of beds in the care homes as a further robustness check. The estimates of the treatment effect will be used in the cost effectiveness analysis, incorporating the uncertainty of the estimates in the analysis.

### Estimating the cost effectiveness of the intervention on health service outcomes

A cost–benefit analysis (CBA) of the intervention will be undertaken. The cost calculation will include both direct and indirect costs to both NHS and care homes delivering the intervention. Benefits are calculated as the change in NHS costs incurred following the intervention. This will be estimated by valuing the reduction in hospital stays and hospital days as a result of the intervention. These will be measured using hospital day rates and ambulance costs. Where possible, data will also be collected on additional costs, such as hospital transfers via taxi and primary care use.

The CBA will be conducted from the perspective of the NHS and Personal Social Services. The costs of the intervention will be compared with the changes in health service costs from reduced hospitalisation. The costs and benefits will be calculated taking account of (1) uncertainty in the estimate of the treatment effect; (2) projected costs over a 5-year period; and (3) spatial variation in cost across jurisdictions. Wherever possible the analytical specification will follow that of the National Institute of Health and Care Excellence (NICE) Reference Case.^44^ While there are also likely to be individual and broader societal benefits arising from the intervention, these are challenging to value in financial terms and beyond the scope of this economic evaluation. They will be explored through the qualitative data collection.

The net benefits of the intervention will be modelled over a 5-year period separately for care homes in (1) England and (2) Scotland, given the estimate of cost savings per care home bed and the total number of care home beds in each jurisdiction, and applying an annual discount rate. These predictions will be modelled at the point estimate for the treatment effect, and also for the upper and lower bounds of the 95% CI around the treatment effect, to provide a range of plausible costs savings over 5 years incorporating the uncertainty in the main study.

The treatment effect will be estimated using a pre and post design. One limitation of this design is that aggregate time trends can be a confounder. Attempts have been made to mitigate this by using multiple sites across the country, and by measuring the baseline and post-treatment outcomes at the same time of year. However, national-level time trends could explain part of the differences observed and this will be reflected on when interpreting the results.

The uncertainty of the estimated treatment effect will be represented in the cost effectiveness analysis. The 95% CIs from the estimated treatment effect will be used to calculate estimated cost effectiveness ranges, that is, the cost effectiveness will be reported at (1) the lower bound of the 95% CI; (2) the point estimate of the treatment effect; and (3) the upper bound of the 95% CI. Reporting a cost effectiveness range will allow the uncertainty in the treatment estimate in the cost effectiveness figures to be captured. Some subgroup analysis is likely to be conducted, for example, to examine cases focused on independent specialist palliative care teams, and public versus private care homes.

### Patient and public involvement

The PPI approach is informed by the National Standards and INVOLVE^45^ guidelines and aims to ensure the study is focused on improving services for residents and families. The PPI representatives were involved from the outset as coapplicants to ensure the research questions were informed by people with lived experience as informal carers of people receiving end-of-life care in care homes. To date, PPI members have informed the choice of family outcome measures^42^ (focusing on a measure which would be least burdensome and most meaningful to relatives), devised interview questions, recruiting the research fellow (RF), contributed to ethical approval documentation and attended the research ethics committee meeting.

PPI members will attend monthly investigator meetings and provide advice on all aspects of the study. They will assist with refining recruitment processes (including recruiting family members to phase 1), participate in the workshops to coproduce Needs Rounds, data collection, and data analysis. The PPI members will also disseminate information about the study and its results through talks to local carers groups and social media (blogs and Twitter). As the project develops, other opportunities for engagement and leadership will be discussed, and PPI members can take on roles which interest them. Bespoke training will be provided to meet any identified needs.

The PPI work will be evaluated throughout and a summative document will be produced at the end of the study. This will include PPI experiences and processes, an audit of PPI resources/costs, and evidence of impact. Interviews will be conducted with PPI members and the research team, including all coinvestigators and representatives from the case study sites. These will examine successes and opportunities to enhance future PPI work. This is likely to be conducted by the study RF, presenting some limitations with objectivity and an independent RF will be used if possible. If sufficient capacity, then a researcher external to the study team will be engaged to increase potential for participants to speak openly about deficits to strengthen the PPI approach.

### Ethics and dissemination

Ethical approval has been granted by the Frenchay REC (287 447) for the implementation study. Separate approval will be sought for the care home survey in 2022.

Participants (table 1) will be provided with a participant information sheet (PIS), including an easy read version for residents. Informed consent will be taken for participation in interviews, questionnaires and Needs Rounds recordings. Care home staff will assess resident capacity and only those who are able to provide informed consent will be included in interviews. Informed consent will not be required for resident data as this will be summary data to ensure anonymity.

Given the sensitive nature of the research, participants may experience distress, particularly residents and relatives. However, the PIS clearly outlines the nature and scope of the study; therefore all participants will be aware of the themes likely to be discussed. Furthermore, the topic guide and questionnaires have been examined by our PPI representatives for appropriateness.

The findings will be disseminated to policy-makers, care home/palliative care practitioners, care home residents/relatives and academic audiences. Infographics, blogs, policy briefings and talks at carer groups/conferences will all be used. An implementation package will be developed for practitioners that provides all the tools and resources required to adopt UK Needs Rounds. The data will be deposited in the open access repository ‘DataSTORRE’.

### Study management and sponsorship

A project steering group will oversee the study. It is chaired by an academic with expertise in care home research and has membership from individuals with expertise in implementation science, health economics, healthcare commissioning, palliative care and PPI.

## Supplementary Material

Reviewer comments
