# Supplementary material for: Palliative and end-of-life care in care homes: protocol for codesigning and implementing an appropriate scalable model of Needs Rounds in the UK
Source: BMJ Open. 2021 Feb 22;11(2):e049486. doi: 10.1136/bmjopen-2021-049486 (PMC7903098; doi:10.1136/bmjopen-2021-049486)
Supplement: Reviewer comments [file bmjopen-2021-049486.reviewer_comments.pdf]

## PEER REVIEW HISTORY

BMJ Open publishes all reviews undertaken for accepted manuscripts. Reviewers are asked to complete a checklist review form (**http://bmjopen.bmj.com/site/about/resources/checklist.pdf**) and are provided with free text boxes to elaborate on their assessment. These free text comments are reproduced below.

## ARTICLE DETAILS

| | |
|---|---|
| **TITLE (PROVISIONAL)** | Palliative and end of life care in care homes: Protocol for co-designing and implementing an appropriate scalable model of Needs Rounds in the United Kingdom |
| **AUTHORS** | Macgregor, Aisha; Rutherford, Alasdair; McCormack, Brendan; Hockley, Jo; Ogden, Margaret; Soulsby, Irene; McKenzie, Maisie; Spilsbury, Karen; Hanratty, Barbara; Forbat, Liz |

This article was not externally reviewed at BMJ Open. Protocols that have been independently assessed prior to submission to BMJ Open are usually fast-tracked to publication on the grounds that further substantial changes will not be possible. This independent assessment will usually be external, independent review for both a substantial grant award from a non-commercial or government funder and ethics approval.
